# A direct referencing method of the tibial plateau for the posterior tibial slope in medial unicompartmental knee arthroplasty

**DOI:** 10.1186/s13018-022-03179-1

**Published:** 2022-06-25

**Authors:** Masao Akagi, Hisafumi Aya, Shigeshi Mori, Nobuhisa Syogaku, Ichiro Tsukamoto, Akihiro Moritake

**Affiliations:** 1 Department of Orthopaedic Surgery, Kindai University Hospital, 377-2 Ohno-Higashi, Osaka-Sayama City, Osaka 589-8511 Japan; 2 Department of Orthopaedic Surgery, Sakura-Kai Hospital, 2610-1 Handa 5, Osaka-Sayama City, Osaka 589-0011 Japan; 3 Department of Orthopaedic Surgery and Rheumatology, Kindai University Nara Hospital, 1248-1 Otoda-cho, Ikoma City, Nara 630-0293 Japan

**Keywords:** A direct referencing method, Medial tibial plateau, Posterior tibial slope, Medial unicompartmental knee arthroplasty, Availability, Accuracy

## Abstract

**Purpose:**

There is no consensus on intraoperative references for the posterior tibial slope (PTS) in medial unicompartmental knee arthroplasty (UKA). An arthroscopic hook probe placed on the medial second quarter of the medial tibial plateau (MTP) in an anteroposterior direction may be used as a direct anatomical reference for the PTS. The purpose of this study is to investigate the availability and accuracy of this method.

**Methods:**

Marginal osteophyte formation and subchondral depression of the MTP and angles between the bony MTP and the cartilage MTP were retrospectively evaluated using preoperative sagittal MRI of 73 knees undergoing medial UKA. In another 36 knees, intraoperative lateral knee radiographs with the probe placed on the MTP were prospectively taken in addition to the preoperative MRI. Then, angles between the bony MTP and the probe axis and angles between the preoperative bony MTP and the postoperative implant MTP were measured.

**Results:**

Among 73 knees, one knee with grade 4 osteoarthritis had a posterior osteophyte higher than the most prominent point of the cartilage MTP. No subchondral depression affected the direct reference of the MTP. The mean angle between the bony MTP and the cartilage MTP was −0.8° ± 0.7° (−2.6°–1.0°, *n* = 72), excluding one knee with a “high” osteophyte. The mean angle between the bony MTP and the probe axis on the intraoperative radiograph was −0.6° ± 0.4° (−1.7–0.0, *n* = 36). The mean angle between the pre- and postoperative MTP was −0.5° ± 1.5° (−2.9°–1.8°). The root-mean-square (RMS) error of these two PTS angles was 1.6° with this method.

**Conclusion:**

Cartilage remnants, osteophyte formation and subchondral bone depression do not affect the direct referencing method in almost all knees for which medial UKA is indicated. When the posterior “high” osteophyte of the MTP is noted on preoperative radiography, preoperative MRI or CT scan is recommended to confirm no “high” osteophyte on the medial second quarter. The accuracy of this method seems equal to that of robotic-assisted surgery (the RMS error in previous reports, 1.6°–1.9°).

## Introduction

Recently, medial unicompartmental knee arthroplasty (UKA) has been recognized as an excellent treatment option for progressive medial knee osteoarthritis (OA) [1–4]. Knee function and patient satisfaction have been reported to be favorably comparable to total knee arthroplasty [5–7]. The functional superiority of medial UKA with nearly normal kinematics is attributable to preservation of functional knee structures, including nondisease compartments and both cruciate ligaments, and restoration of the preoperative joint surface morphology and knee alignment [8–11]. Conversely, proper implant positioning is critical for long-term survival after UKA, and malpositioning and alignment can alter the biomechanics of the knee, increasing ligament strain and contact stresses [12–14].

The posterior tibial slope (PTS) of the medial tibial plateau (MTP), defined as the posterior inclination of the plateau relative to the tibial bone axis, is considered an important anatomical feature that influences cruciate ligament function and sagittal plane stability of the knee [15–19], and wide individual variations of up to 14° have been reported in the PTS for both normal and arthritic knees [20–22]. PTS has been reported to affect implant survival [20], postoperative range of motion [19, 23], and knee kinematics after UKA [10, 24]. Therefore, accurate recreation of the preoperative PTS is critical for the optimization of postoperative knee function and longevity [20].

In the operative field of modern mini-incision UKA, however, it is difficult to observe the entire MTP, including its posterior edge, until completion of the femoral bony preparation. Recently, using preoperative CT data in patients with medial UKA, we have suggested that we refer the MTP to recreate the native PTS by placing an arthroscopic probe in the anteroposterior (AP) direction on the medial second quarter of the MTP [25]. However, deformities of the MTP associated with OA progression, including cartilage remnants and cartilaginous osteophyte formation, may make it difficult to use the MTP itself as an anatomical reference. In this study, preoperative sagittal magnetic resonance imaging (MRI) in knees undergoing medial UKA was retrospectively reviewed to evaluate the influence of OA changes on the MTP. Furthermore, intraoperative lateral knee radiographs with a hook probe placed on the MTP and postoperative lateral knee radiographs were prospectively taken. The purpose of this study is to demonstrate the availability and accuracy of the direct referencing method of the MTP in medial UKA.

## Patients and methods

### Patients

#### Study I: Preoperative MRI studies

Preoperative MRI examination of the knee is routinely performed in our institute to check the conditions of the anterior cruciate ligament (ACL), the lateral femorotibial compartment and the patellofemoral joint and to determine indications for UKA. In this study, 73 consecutive knees with primary medial UKA between June 2018 and July 2020 were included. Surgical indications for medial UKA included medial unicompartmental knee OA, a functional ACL, correctable varus deformity and flexion deformity of < 15°. Inflammatory arthritis and severe knee OA with strong destruction of the MTP and/or subluxation of the femorotibial joint were excluded from indication of medial UKA. The mean age of the patients was 76.5 ± 5.8 years (62–86), and there were 16 male and 57 female knees. The severity of OA according to the K–L classification [26] was grades 2, 3 and 4 in 13, 46 and 14 knees, respectively. The mean femorotibial angle (FTA) in the standing AP radiographs was 181.4° ± 2.7° (175–186), and the mean preoperative PTS was 8.3° ± 1.8° (3.4–15.4).

#### Study II: Intra- and postoperative lateral knee radiograph studies

Between February 2021 and January 2022, 36 consecutive knees were considered to have indications for medial UKA and were prospectively collected to confirm the results of Study I. Preoperative MRI examination of the knee was performed for all patients as in Study I. The mean age of the patients was 75.0 ± 8.9 years (range, 48–88 years), and there were 7 male and 29 female knees. The severity of OA was grade 2, 3 and 4 in 6, 22 and 8 patients, respectively. The mean FTA on standing AP radiographs was 180.1° ± 2.6° (177–185), and the mean PTS was 8.5 ± 2.4° (3.3–14.1).

Both the Study I and II protocols were approved by the Institutional Review Board, and informed consent was obtained from each patient prior to study enrollment.

### Imaging technique

The knee MRI in Studies I and II was performed using a 1.5-Tesla imager (Signa HDxT, General Electric Medical Systems, Milwaukee, WI) with an eight-channel knee coil (HD TR knee phase array). The patients were positioned supine on the MRI scanning table with the knee extended and the patella facing upward. Coronal, sagittal and axial images were obtained in a standardized manner. Images were acquired according to a common standard knee MRI protocol of the Department of Radiology, which included coronal and sagittal multiple echo recalled gradient echo images for the present study (TR: Cor/Sag 800/600, each with 4 echoes). A 3.5-mm slice thickness with a 0.5-mm slice space was used.

The intraoperative lateral knee radiograph was taken using a portable X-ray machine (MobileDaRt Evolution MX8 Version, SHIMADZU, Kyoto, Japan), which has wireless flat panel detectors (FPDs, DR-ID800, FUJIFILM, Tokyo, Japan) and can show reference X-ray images on an installed monitor immediately after radiography. After arthrotomy, stripping off of the medial capsular attachment from the proximal tibia, and resection of the anterior half of the medial meniscus, an arthroscopic hook probe was placed on the medial second quarter of the MTP (between the medial 1/4 and 1/2 MTP), parallel to the AP tibial line [27, 28] with the knee flexed at approximately 90°. Radiography was repeated until the correct laterality was confirmed on the monitor of the portable machine.

### Evaluation of osteophyte height and expansion of subchondral bone depression in the MTP using sagittal MRI

**Fig. 1:**
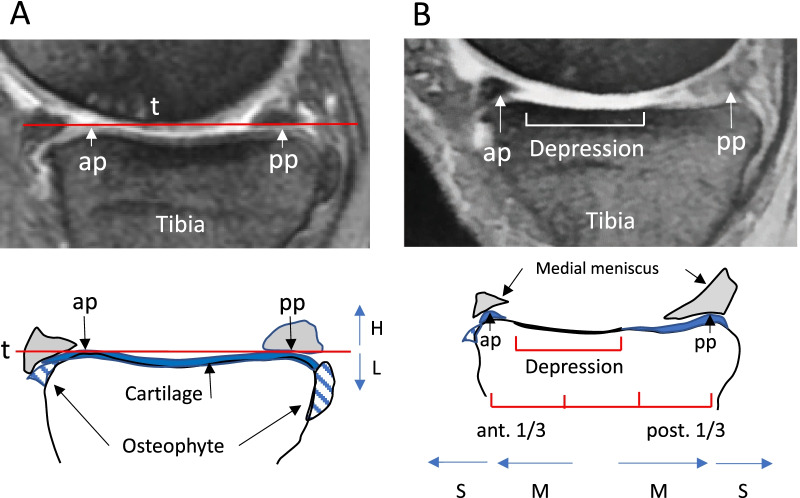
Evaluation of the height of the anterior/posterior osteophyte and expansion of anterior/posterior subchondral bone depression (attrition) on the medial second quarter of the MTP using sagittal MRI. **A** Osteophyte height is classified into three categories (N: none, L: low, H: high) relative to a line t. **B** Expansion of the depression is classified into three categories (N: none, M: moderate, S: severe) based on location. ap: anterior most prominent aspect of the MTP, pp: posterior most prominent aspect of the MTP, t: a line tangent to the ap and pp

Using sagittal MRI, we evaluated the height of the anterior/posterior osteophyte and anterior/posterior expansion of subchondral bone depression (attrition) [29] on the medial second quarter of the MTP to determine whether they can affect the MTP indicated by the arthroscopic probe placed on it. Relative to a line tangent to the most prominent aspects of the anterior and posterior cortices of the MTP including cartilage remnants (line t in Fig. 1A), osteophyte height was classified into three categories as follows: none (N), there is no osteophyte; low (L), same as or lower than the line t; and high (H), higher than line t. Based on location, expansion of the subchondral depression was classified into three categories as follows: none (N), there is no depression; moderate (M), the depression expands to the anterior or the posterior 1/3 of the MTP but does not reach the most prominent aspect of the anterior and posterior cortices; and severe (S), the depression expands to or over the most prominent aspects of the anterior or posterior cortices (Fig. 1B).

### Angular measurements on sagittal MRI

**Fig. 2:**
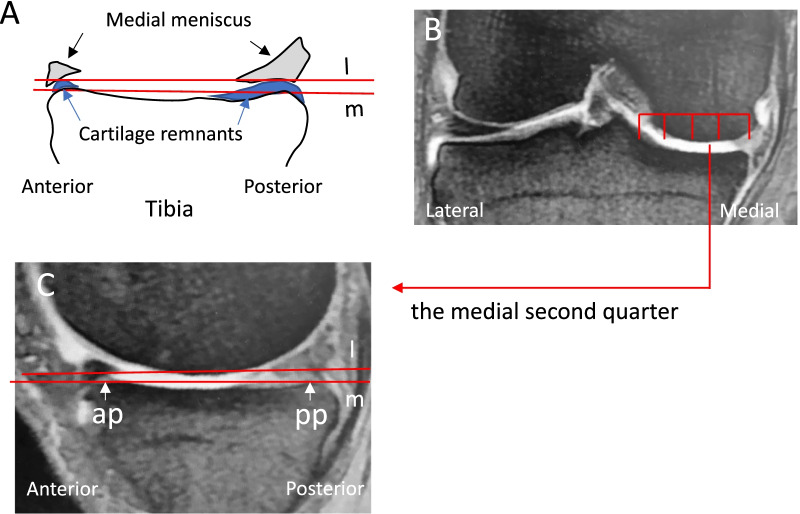
Measurement of the angles on sagittal knee MRI. **A** The preoperative bony slope of the MTP was defined as a line tangent to the most prominent aspects of the anterior and posterior cortices of the MTP (line m), and the cartilage slope of the MTP was defined as a line tangent to the most prominent aspects of the anterior and posterior cartilage remnants (line l). **B** The frontal view of the knee MRI, in which the MTP is divided into four parts. **C** On the medial second quarter of the MTP, the angle between lines m and l is measured. ap: anterior most prominent aspect of the MTP pp: posterior most prominent aspect of the MTP

The preoperative bony MTP in sagittal MRI was defined as a line tangent to the most prominent aspects of the anterior and posterior cortices (subchondral bone plate) according to methods previously described [24, 30] (line m in Fig. 2A and C). The cartilage slope of the MTP was defined as a line tangent to the most prominent aspects of the anterior and posterior cartilage remnants of the MTP (line l in Fig. 2A and C). Frontal and sagittal views of the knee MRI were simultaneously displayed on an image analysis monitor. While the frontal view showed a middle slice of the knee (Fig. 2B), a slice of the medial second quarter of the MTP (Fig. 2B) was displayed on the sagittal view. The angle formed by the two tangential lines (lines m and l) was measured on both slices using image analysis software (PACS system FABRICA Ver. 1.0.0.23, Cure Hope Corp., Osaka, Japan). A positive value was given to the angle measurement when the cartilage PTS (line l) was larger than the preoperative bony PTS (line m).

### Surgical technique with the direct referencing method

All surgical procedures in Study II were performed by one senior surgeon (M.A.), and a fixed-bearing UKA implant (Tribrid UKA system, Kyocera Corp., Kyoto, Japan) was implanted through the medial mini-parapatellar skin incision. The operation was performed using the so-called tibia-cut first and spacer block technique. First, the substitute tibial AP line [28] was drawn to pass through the medial tibial eminence and the medial edge of the patellar tendon at the joint level. Then, an arthroscopic hook probe was placed in the AP direction on the medial second quarter of the MTP. An individual implant PTS was determined by setting a gauge inserted into the cut slot of the extramedullary guide parallel to the probe (see Fig. 7E).

### Angular measurements on the intra- and postoperative lateral knee radiographs

**Fig. 3:**
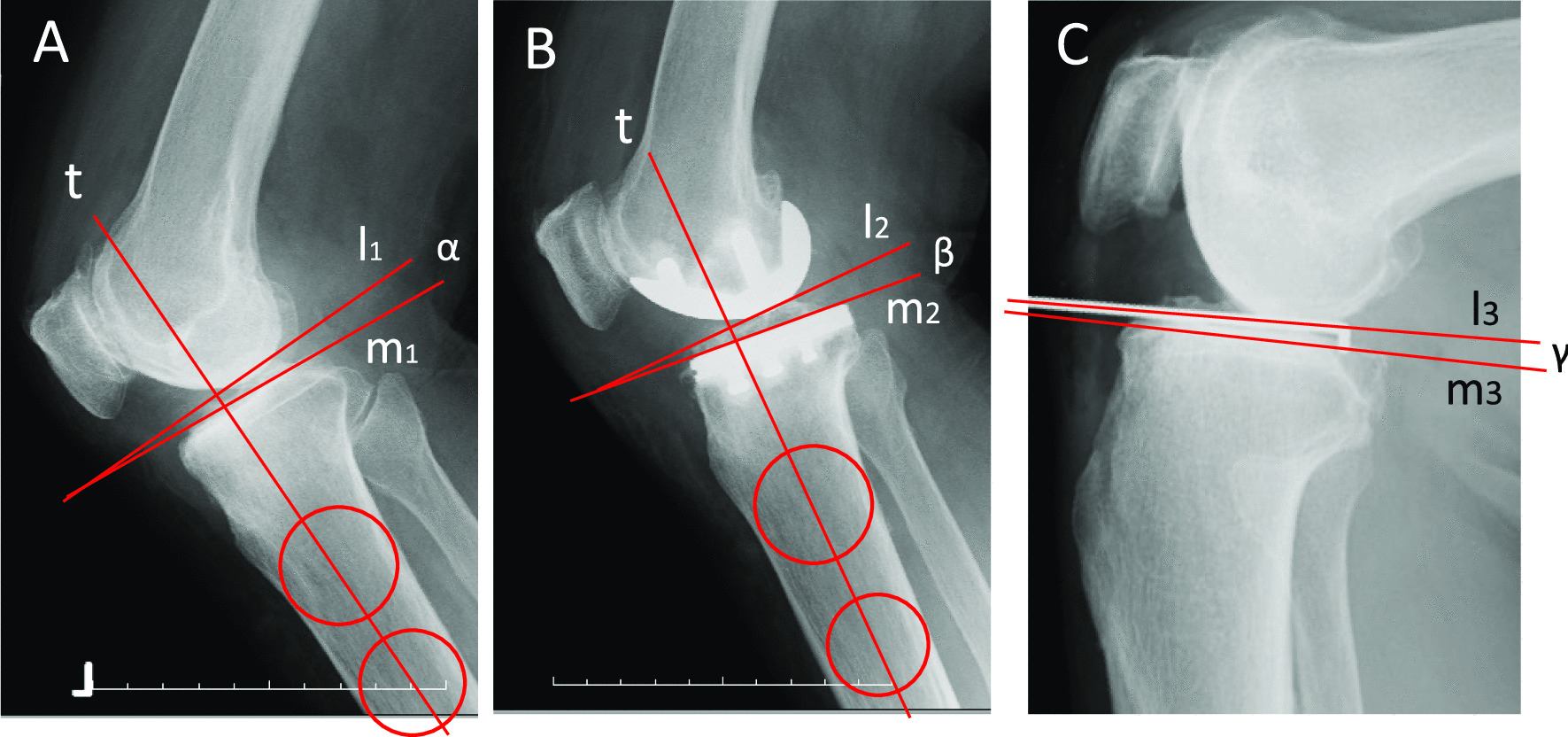
Measurement of the angles on preoperative, intraoperative, and postoperative lateral knee radiography. Preoperative bony PTS (α) and postoperative implant PTS (β) on a lateral radiograph were measured according to a method previously reported (**A, B**). On the intraoperative lateral knee radiograph, an angle (γ) between the native bony MTP (m_3_) and the cartilage MTP (l_3_) was measured (**C**)

Preoperative bony PTS (α) and postoperative implant PTS (β) on a lateral radiograph were measured according to a method previously reported [20]. A line passing through the center of 2 circles located over the AP width of the tibia was drawn (line t in Fig. 3A and B). Then, a line perpendicular to line t was drawn (line l_1_). The angle between line l_1_ and a line tangential to the subchondral plate of the MTP (line m_1_) was measured as the preoperative bony PTS (angle α in Fig. 3A). Similarly, implant PTS was defined as an angle between a line l_2_ and a line m_2_ tangential to the upper surface of the tibial tray (angle β in Fig. 3B). On the intraoperative lateral knee radiograph, the native bony MTP was defined as a line tangent to the subchondral plate of the MTP (line m_3_). The cartilage slope of the MTP was defined as a line of the probe axis placed on the medial second quarter of the MTP with cartilage remnants (line l_3_). The angle formed by these two lines was measured (angle γ in Fig. 3C).

### Statistical analysis

The height of the osteophyte and expansion of subchondral bone depression in each knee were classified by two observers (M.A. and H.A.), upon their discussion and confirmation. All angular measurements on the sagittal MRI and lateral knee radiography were independently performed twice by two observers (S.M. and N.S.), and the mean of four measurements was considered a true value. The intraclass correlation coefficient for interrater reliability of these two observers on the MRI and on the intraoperative lateral knee radiography was 0.68 and 0.77, respectively. The results are presented as the mean ± standard deviation (SD) and were processed using Microsoft Excel 2016 (Microsoft Corp., Redmond, WA). Differences between results were evaluated using Student’s unpaired or paired *t* tests. Pearson’s correlational analysis was performed to analyze the relationships between the two angle measurements in Study II. The root-mean-square (RMS) error was used to evaluate the accuracy of the direct referencing method.

## Results

### Study I: Height of the anterior/posterior marginal osteophyte (Fig. 4A)

Eighty-four and 66% of total knees had an anterior “low” and a posterior “low” osteophyte, respectively. In 14 knees with Grade 4 OA, one knee (7%) had a “high” posterior osteophyte. Although the number of knees with anterior and/or posterior osteophytes increased with the K-L grade, the number of knees with anterior osteophytes increased from the lower grade compared with the number of knees with posterior osteophytes.

**Fig. 4:**
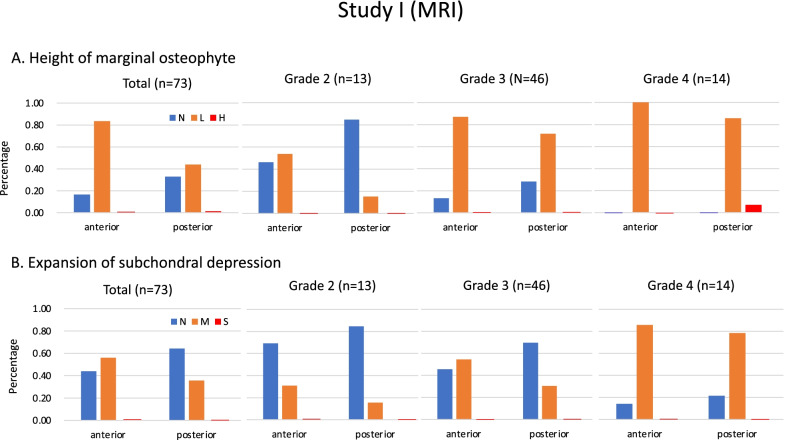
Percentage of the knee classified according to the categories in the total (*n* = 73), Grade 2 (*n* = 13), Grade 3 (*n* = 46) and Grade 4 (*n* = 14). A height of marginal osteophytes, which is classified into three categories (N: none, L: low, H: high). Only one case in Grade 4 had a “high” posterior osteophyte among patients undergoing medial UKA in Study I. B Expansion of subchondral bone depression, which was classified into three categories (N: none, M: moderate, S: severe). There were no cases in which the anterior or posterior subchondral depression expanded over the most prominent aspect of the anterior or posterior cortices, respectively

### Study I: Expansion of subchondral bone depression (Fig. 4B)

Fifty-six and 37% of the total knees had anterior “moderate” and posterior “moderate” depression, respectively. Although knees with anterior and/or posterior depression increased with the K-L grade, the number of knees with anterior depression increased from the lower grade compared with the number of knees with posterior depression. There was no knee with anterior and/or posterior “severe” depression in these 73 knees.

### Angular measurements in the sagittal view of the MRI in Study I (Fig. 5)

**Fig. 5:**
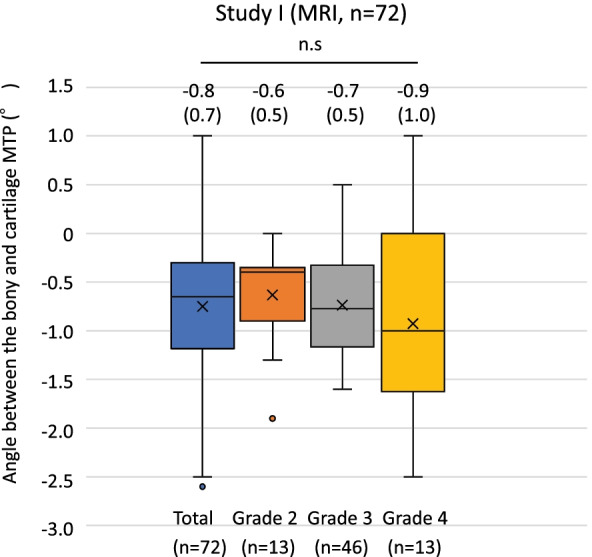
Box plots showing angles between the native bony MTP (line m in Fig. 2) and the cartilage MTP (line l in Fig. 2) in the total (*n* = 72), Grade 2 (*n* = 13), Grade 3 (*n* = 46) and Grade 4 (*n* = 13) of Study I. There was no significant difference between the mean angles of Grades 2, 3 and 4

Angular measurements were performed for all knees except for one knee with a posterior “high” osteophyte. The mean angle of the total knees between the two tangential lines (line m and line l in Fig. 2) on the sagittal view of MRI was −0.8° ± 0.7° (−2.6°–1.0°, *n* = 72). There was no significant difference between the mean angle in Grade 2 (−0.6° ± 0.5°, −1.9°–0.0°, *n* = 13), Grade 3 (−0.7° ± 0.5°, −1.6°–0.5°, *n* = 46), and Grade 4 (−0.9° ± 1.1°, −2.5°–1.0°, *n* = 13) (Fig. 5). The angle between the two tangential lines in one knee with the posterior “high” osteophyte was 3.2 degrees.

### Angular measurements on the preoperative sagittal MRI and on the intra- and postoperative lateral knee radiographs in Study II

**Fig. 6:**
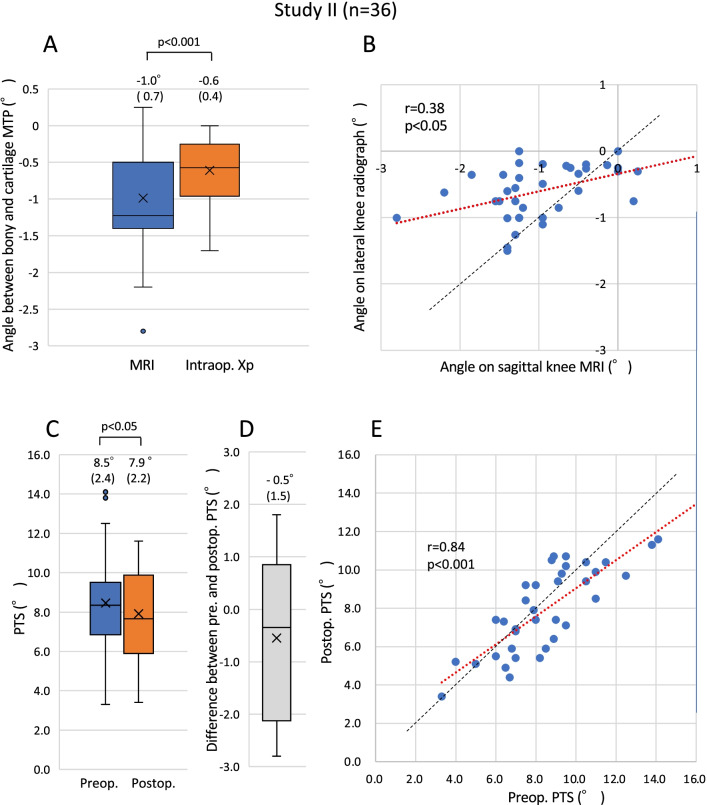
Findings in Study II (*n* = 36). **A** Box plots showing angles between the bony MTP and the cartilage MTP on preoperative sagittal MRI and intraoperative lateral knee radiographs. The absolute value of the mean angle on the intraoperative radiograph was significantly smaller than that on the MRI. **B** A scatter diagram of the angles on the preoperative sagittal MRI and the intraoperative lateral knee radiograph. There was a week but significant correlation between them (*r* = 0.38, *p* < 0.05). In many cases, the angle on the intraoperative radiograph was smaller than that on the preoperative MRI. **C** Box plots showing preoperative boney PTS and postoperative implant PTS. **D** A box plot showing the difference between pre- and postoperative PTS. E. A scatter diagram of the angles of pre- and postoperative PTS. A strong correlation was noted (*r* = 0.84, *p* < 0.001)

There was no knee with anterior and/or posterior “high” osteophytes in Study II. Furthermore, there was no knee with anterior and/or posterior “severe” depression. The mean angle between the two tangential lines (lines m and l in Fig. 2) on the preoperative sagittal MRI was −1.0° ± 0.7° (−2.8–0.3, *n* = 36). The mean angle between the probe axis and the bony MTP on the intraoperative lateral knee radiograph (lines m_3_ and l_3_ in Fig. 3C) was −0.6° ± 0.4° (−1.7–0.0, *n* = 36). The mean angle on the intraoperative lateral knee radiograph was smaller than that on the sagittal MRI (*p* < 0.001, paired *t* test, *n* = 36, Fig. 6A). A weak but significant correlation between the angles on the sagittal MRI and on the lateral knee radiograph was observed (*r* = 0.38, *p* < 0.05, Fig. 6B). The mean angles of the preoperative PTS and the implant PTS were 8.5° ± 2.4 (3.3–14.1) and 7.9° ± 2.2° (3.4–11.6), respectively, and there was a significant difference between these mean angles (*p* < 0.05, paired *t* test, *n* = 36, Fig. 6C). The mean angle difference between the preoperative and implant PTS was −0.5° ± 1.5° (−2.8–1.8) (Fig. 6D). The RMS error of the tibial slope was 1.6° with this method. There was a strong correlation between the preoperative and implant PTS (*r* = 0.83, *p* < 0.001, Fig. 6E).

### Case presentation

**Fig. 7:**
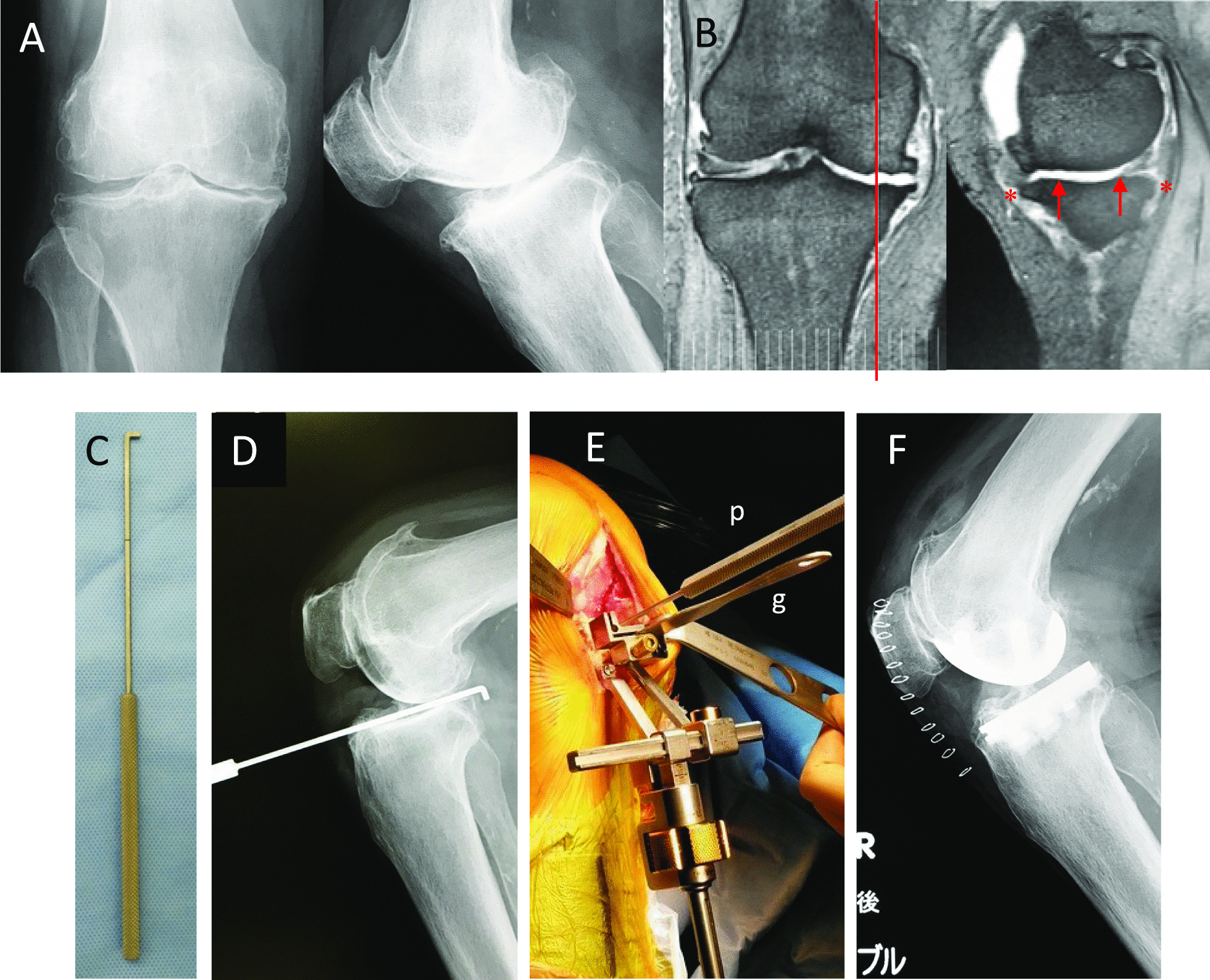
Case presentation. **A** Preoperative knee radiographs with Grade 4 OA. **B** Preoperative MRI. A red line: a slice line on the medial second quarter of the MTP. *: anterior and posterior “low” osteophyte. ↑: anterior and posterior “mild” depression on the MTP. **C** A hook probe. **D** An intraoperative lateral knee radiograph with the probe on the medial second quarter. **E** A photograph showing how to set the extramedullary cutting guide in this method. g: A gauge inserted into the slot of the tibial cut block. p: the probe. **F** Postoperative lateral knee radiograph. The native bony PTS is recreated

An 88-year-old male patient presented with grade 4 knee OA (Fig. 7A). Preoperative MRI showed anterior and posterior “low” osteophytes (*) and anterior and posterior “mild” depression on the MTP (Fig. 7B). A hook probe (Fig. 7C) was placed on the medial second quarter of the MTP in an AP direction. Then, an intraoperative lateral knee radiograph was taken (Fig. 7D). A gauge (g in Fig. 7E) inserted into the slot of the tibial cut block was set parallel to the probe (p in Fig. 7E). A postoperative lateral knee radiograph demonstrated that the native bony PTS was recreated (Fig. 7F).

## Discussion

The results of Study I indicated that a knee with a “high” osteophyte on the medial second quarter of the MTP, which can affect the direct referencing method, is rare among knees with indications for medial UKA. Among 73 knees in Study I, only one knee with Grade 4 OA had a “high” posterior osteophyte. Furthermore, among 36 knees in Study II, no knee had a “high” osteophyte on the medial second quarter of the MTP. Observing all sagittal slices of the MRI, posterior osteophytes higher than the most prominent aspect of the cartilage MTP were observed near the posterior cruciate ligament attachment in a small number of knees with Grade 4 OA. This observation is consistent with that of Mullis et al. [31], who stated that the posterior osteophyte of the MTP in varus knee OA, which is sometimes noted on the lateral knee radiograph, is intraoperatively observed at the posterolateral margin of the MTP. In addition, in the medial second quarter, there were no cases in which the anterior or posterior subchondral depression expanded over the most prominent aspect of the anterior and/or posterior cortices of the MTP. Therefore, it seems rare that osteophytes and subchondral depression affect the direct referencing method of the MTP in knees for which medial UKA is indicated.

In both Studies I and II using MRI, the cartilage slope of the MTP was slightly smaller than the preoperative bony slope in many knees. This may be explained by the anteromedial cartilage wear on the MTP in early- to moderate-stage knee OA with a functional ACL [32, 33]. This angular error on the MRI was estimated to be 1.0° on average and less than 2.5° and is considered negligible in clinical practice because it would be virtually impossible to manually adjust the extramedullary tibial cutting guide for less than single-degree precision. A small decline in the PTS may keep the bone stock in the proximal tibia and reduce the risk of increased postoperative stress on the ACL [16, 20].

In Study II, the mean angle between the bony MTP and the cartilage (probe axis) MTP on intraoperative lateral knee radiography was smaller than that on preoperative sagittal MRI. In addition, the correlation between these two angles was significant but weaker than expected. The hook probe inserted into the tight joint space may be pressed into the cartilage remnants and placed close to the bony MTP, which might have made the angle on intraoperative lateral knee radiograph smaller than the angle on the preoperative sagittal MRI and made the correlation between these two angles weak.

We prospectively investigated the accuracy of the direct referencing method in Study II. The postoperative implant PTS significantly decreased by 0.5° on average compared with the preoperative bony PTS. This decline could be attributable to the difference between the bony MTP and the cartilage MTP demonstrated in Studies I and II. The RMS error between the planned and achieved PTS has been reported to be 1.6° to 1.9° with robotic assistance [34, 35]. Bush AN et al. reported that the RMS error for PTS relative to the surgeon’s goals was 1.5° when an experienced surgeon manually performed medial UKA [36]. In this study, the RMS error of the PTS was 1.6° with the direct referencing method. Furthermore, a strong correlation between the preoperative bony PTS and the implant PTS was found. The accuracy of this method is considered to be equal to that of robotic-assisted surgery or surgery by an experienced high-volume surgeon.

The present study had certain limitations. First, the study population included only Japanese patients. Furthermore, the indication for medial UKA for the patients enrolled in the present study was determined by one surgeon (M.A.). Caution may be needed when the clinical application of our findings is considered in other nations and is adopted by other surgeons. Second, caution should be exercised regarding the flexibility of the arthroscopic probe. If the probe is thin and flexible, the axis of the probe can indicate a larger PTS than the preoperative bony PTS due to the bend of the probe. Third, it is rare, but there was one knee with Grade 4 OA that had posterior “high” osteophytes on the medial second quarter of the MTP. When the posterior “high” osteophyte is noted on the preoperative lateral knee radiograph, preoperative MRI or CT scan is recommended to confirm no “high” osteophyte on the medial second quarter of the MTP.

## Conclusions

Cartilage remnants, osteophyte formation and subchondral bone depression do not affect the direct referencing method of the MTP, and the direct referencing method is available in almost all knees for which medial UKA is indicated. When a posterior “high” osteophyte on a preoperative lateral knee radiograph is noted, preoperative MRI or CT scan is recommended. The accuracy of this method seems to be equal to that of robotic-assisted surgery or surgery performed by experienced high-volume surgeons, although the probe on the MTP would indicate the PTS slightly smaller than the native bony PTS.

## Data Availability

The datasets used or analyzed in the current study are available from the corresponding author on reasonable request.

## Abbreviations

UKA: Unicompartmental knee arthroplasty
OA: Osteoarthritis
PTS: Posterior tibial slope
MTP: Medial tibial plateau
AP: Anteroposterior
MRI: Magnetic resonance imaging
ACL: Anterior cruciate ligament
FTA: Femorotibial angle
SD: Standard deviation
RMS: Root mean square
